# Sensitization to PR-10 proteins is indicative of distinctive sensitization patterns in adults with a suspected food allergy

**DOI:** 10.1186/s13601-017-0177-4

**Published:** 2017-11-23

**Authors:** Mark A. Blankestijn, André C. Knulst, Edward F. Knol, Thuy-My Le, Heike Rockmann, Henny G. Otten, Rob J. B. Klemans

**Affiliations:** 1 Department of Dermatology and Allergology, University Medical Center Utrecht, Utrecht University, Heidelberglaan 100, 3584 CX Utrecht, The Netherlands; 2 Laboratory of Translational Immunology, University Medical Center Utrecht, Utrecht University, Heidelberglaan 100, 3584 CX Utrecht, The Netherlands

**Keywords:** ImmunoCAP ISAC, Food allergy, PR-10, Specific IgE, Storage proteins

## Abstract

**Background:**

The extent of co-sensitization within and between food protein families in an adult population is largely unknown. This study aimed to identify the most frequently recognized components in the PR-10 and storage protein family, as well as patterns in (co-)sensitization, in a birch-endemic area.

**Methods:**

Results of ImmunoCAP ISAC, performed during routine care in Dutch adult outpatients suspected of food allergy, were collected.

**Results:**

A total of 305 patients were selected, aged 16–79 years (median 32 years). Sensitization to one or more PR-10 proteins was most frequent (74% of all subjects), followed by 35% to storage protein and 15% to nsLTPs. Within the PR-10 family, subjects were most often sensitized to Bet v 1 (73% of 305), Cor a 1.04 (72%) and Mal d 1 (68%). Sensitization to PR-10s from soy, celery and kiwi occurred distinctively less often (< 55% of Bet v 1 sensitized subjects) compared to other food PR-10s (all > 70%). Subjects sensitized to these ‘less common PR-10 proteins’ were sensitized to more food and inhalant components on the ISAC, compared to subjects sensitized to ‘common PR-10 proteins’ (median 22 vs 13 out of 112, *p* < 0.0001). Seven subjects demonstrated sensitization to food PR-10 proteins, without concomitant sensitization to pollen PR-10s. Within the storage proteins, sensitization to multiple peanut allergens was most common (on average 3 out of 4).

**Conclusions:**

Sensitization to PR-10 food proteins could occur without concomitant sensitization to common PR-10 from pollen in a subset of subjects. Less commonly recognized PR-10 proteins appear to be an indication of polysensitization.

**Electronic supplementary material:**

The online version of this article (10.1186/s13601-017-0177-4) contains supplementary material, which is available to authorized users.

## Background

In food allergy diagnostics, component-resolved diagnostics (CRD) allows the clinician to assess the presence of specific IgE (sIgE) to allergenic proteins (components), instead of crude extracts. A consensus document by the WAO-ARIA-GA2LEN Task Force elaborates the role of CRD in three major aspects of allergy diagnostics: distinguishing between genuine versus cross-reactive sensitization, assessing the possible risk of a severe allergic reaction upon exposure in selected patients and identifying allergens for specific immunotherapy [1]. The ImmunoCAP Immuno-Solid phase Allergen Chip (ISAC; Thermo Fisher Scientific, Uppsala, Sweden) is a multiplex technology to assess the presence of specific IgE to a large number of components at the same time [2]. Studies have demonstrated a good concordance between the ImmunoCAP and ImmunoCAP ISAC in assessing sensitization to components [3, 4]. The current ISAC model holds 112 components, which are a selection of known food and inhalant allergens, as well as allergens from others sources such as latex (see Additional file 1: Table 1).

Components can be grouped into protein families based on their biological function and structural homology [5–7]. In plant food allergy, important protein families include the pathogenesis related protein family 10 (PR-10) proteins, profilins, seed storage proteins and non-specific lipid transfer proteins (nsLTP). PR-10 proteins and profilins are considered labile proteins, and sensitization to these proteins is usually associated with mild symptoms, mostly limited to the oral cavity [8]. Storage proteins and nsLTPs on the other hand, are considered resistant to thermal processing, and pH changes, and are associated with moderate to severe symptoms upon ingestion [5, 9–13]. The amount of cross-reactivity between allergens from the same family varies between protein families. Extensive cross-reactivity is seen within the PR-10 proteins and profilins [14]. Clinical cross-reactivity between storage proteins of different plant foods appears to be limited, although (in vitro) cross-reactivity within and between specific tree nuts and legumes has been described [15–17]. Co-sensitization, also between different protein families, is a common phenomenon in daily practice [18].

The aim of this paper was to identify the most frequently recognized plant food components and protein families using sensitization data from multiplex CRD in an adult population suspected of food allergy. Additionally, we wanted to investigate (co-)sensitization patterns with regard to food protein families, focused on PR-10 and storage proteins. For this purpose, we focused on sensitization only, while also including demographic data such as age and sex.

## Methods

### Patient selection

A retrospective explorative study was conducted. The study population consisted of every adult patient that received diagnostic analysis using ImmunoCAP ISAC 112 in our food allergy outpatient clinic of the Dermatology/Allergology department of our tertiary hospital in Utrecht, The Netherlands, between April 2012 and September 2016. All results of the ImmunoCAP ISAC were collected, regardless of clinical parameters. In case more than one ISAC was performed in a single patient, which was the case in *n* = 3, only the first/oldest was included. The population consisted of patients referred by general practitioners and secondary care dermatology and allergy clinics because of a potential food allergy. In general, an ISAC was ordered based on the physician’s judgment in case of suspicion of multiple (plant) food allergies (i.e. fruits, legumes and tree nuts). Demographic data, i.e. age at time of blood draw and sex, were acquired for each patient.

### ImmunoCAP ISAC

The ImmunoCAP ISAC was performed according to the manufacturer’s instructions (Thermo Fisher Scientific, Uppsala, Sweden). On the ISAC, specific IgE binds to the components spotted on the chip and is then detected by immunofluorescence. Fluorescence intensity is measured by laser scanning and reported semi-quantitatively as ISAC Standardized Units (ISU) [19]. ISU values are reported in a range of 0.3–100. A patient was considered sensitized to a component on the ISAC in case of an ISAC Standardized Units (ISU) value of 0.3 or higher as recommended by the manufacturer. Subjects with sIgE against nJug r 2 were considered truly sensitized to Jug r 2 in case of no detectable sIgE against CCD marker nMUXF3 on ISAC, excluding reactivity to known carbohydrate epitopes on nJug r 2 on ISAC [20].

### Statistics

Descriptive analyses were performed and presented as percentages or median values with interquartile range (IQR). The Chi square test was performed to assess differences in sex or sensitization frequency between groups. Differences in age and ISU levels between groups were assessed by Mann–Whitney *U* test. Spearman correlation was used to analyze correlations between ISU levels. A *p* value < 0.05 was considered statistically significant. Where necessary, a Bonferroni correction was applied to correct for multiple testing (112 components) by using a *p* value of 0.0004. Analyses were performed using SPSS Statistics 21 (IBM Corporation, Armonk, NY, USA).

### Ethics

All ISAC tests were performed during routine care. The local Medical Ethics Review Committee confirmed that ethical approval is not required (nr. 15-249).

## Results

A total of 305 patients with ISACs performed were included. Median age of the subjects was 32 years (range 16–79, IQR 22–45) and 74% was female. Sixteen ISACs (5%) were negative for all tested components. The following analyses were done using the total population of 305 subjects, unless stated otherwise. In our population, 91% was sensitized to two or more components of the ISAC and 85% to five or more, with a median amount of components recognized of 17 out of 112.

**Fig. 1:**
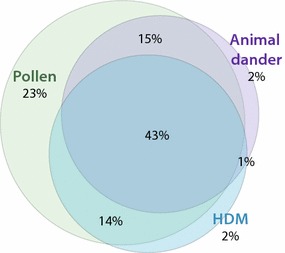
Co-sensitization in subjects sensitized to any pollen (tree, grass and weed), house dust mite (HDM) and animal dander (cat, dog, mouse or horse) proteins. Of the total population (*n* = 305), 25 subjects were not sensitized to any component of the three inhalant allergen groups

Out of 305 subjects, 92% was sensitized to at least one inhalant component on the ISAC. The major inhalant allergens include pollen (tree, grass and weed), house dust mite (HDM) and animal dander (cat, dog, mouse or horse). Tree pollen sensitization was most common (79%), with birch, alder and hazel pollen’s PR-10 proteins being the most often recognized (see Additional file 1: Table 1), followed by grass (70%) and weed pollen (32%). Of our population, 55% recognized at least one component of the common Dermatophagoides house dust mites (Der p 1, Der p 2, Der p 10, Der f 1 and Der f 2). Sensitization to animal dander was seen in 56% of all subjects, with cat dander being most often recognized (49%). Polysensitization to inhalant allergens was very common with 73% sensitized to two or more of the inhalant allergen groups (pollen, HDM, animal dander) (Fig. 1). Of all subjects, 43% was sensitized to components from all three groups (pollen, HDM, animal dander; Fig. 1).

In all subjects sensitized to at least one food component (*n* = 265; 87%), the median amount of food components recognized was 6 out of 49. Additionally, 87% of the 265 was sensitized to three or more food components and 15% to 10 or more. The top recognized components were Bet v 1, Cor a 1.04 and Mal d 1 (see Additional file 1: Table 1). Of the total population, 74% was sensitized to any food PR-10 protein, followed by 35% to any storage protein, 15% to any food nsLTP, 8% to food tropomyosin (Pen m 1) and 3% to parvalbumin (Gad c 1). Since no food components from the profilin family are present on the ISAC, sensitization to Birch pollen profilin Bet v 2 was used as an indication for profilin sensitization [21], which was observed in 15% of all subjects. The following results will focus on PR-10 and storage protein sensitization since they were the most recognized plant food allergens.

### Co-sensitization between protein families

**Fig. 2:**
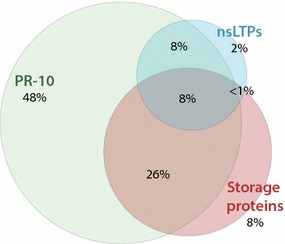
Co-sensitization in subjects sensitized to at least one food PR-10 protein, storage protein or non-specific lipid transfer protein (nsLTPs) from food, present on the ImmunoCAP ISAC. Of the total population (*n* = 305), 52 subjects were not sensitized to any food component of the three protein families

Sensitization often was not limited to just pollen or non-pollen related food components. Of all patients sensitized to one or more PR-10 food components, 34% was also sensitized to one or more storage proteins. The overlap of sensitization to PR-10 proteins, storage proteins and nsLTPs, the third most recognized protein family of food components, is shown in Fig. 2.

### Sensitization to food PR-10 proteins without pollen sensitization

Seven of the 228 subjects sensitized to PR-10s (3%) demonstrated sensitization to food PR-10 components without concomitant sensitization to PR-10 pollen components from birch (Bet v 1), alder (Aln g 1) or hazel (Cor a 1.01). Of these seven subjects, three were only sensitized to Cor a 1.04, three to Ara h 8 and one to both Cor a 1.04 and Ara h 8. In these subjects, titers for the positive PR-10 food components were generally low (< 2 ISU). Interestingly, all four subjects sensitized to Ara h 8 were also sensitized to Ara h 1, 2, 3 and 6, but not Ara h 9. Of the four sensitized to Cor a 1.04, only one demonstrated sensitization to other hazelnut components.

### Sensitization patterns within PR-10 proteins

**Fig. 3:**
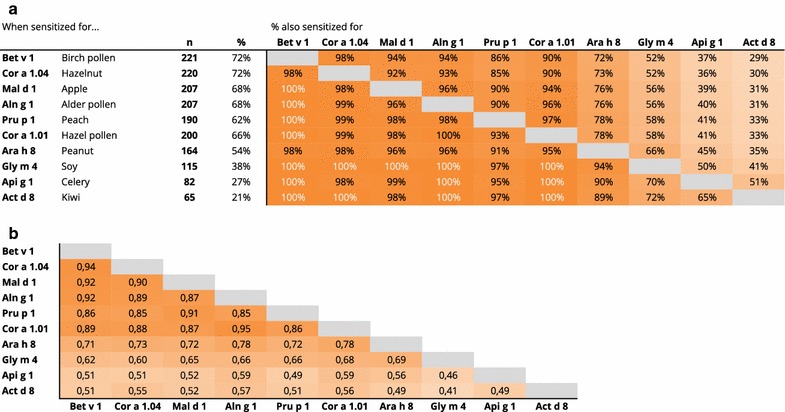
**a** Frequency of sensitization to PR-10 proteins (left) and percentage of sensitized subjects with concomitant co-sensitization to other PR-10 proteins (right). Darker shade of orange indicates higher frequency of co-sensitization. **b** Spearman correlation results of the ISU values found for the different PR-10 proteins

Subjects sensitized to PR-10 proteins (*n* = 228) were in most cases (≥ 90%) sensitized to a combination of at least six PR-10 proteins: Bet v 1, Cor a 1.04, Mal d 1, Aln g 1, Pru p 1, Cor a 1.01 (Fig. 3a). Sensitization to PR-10 proteins Gly m 4, Api g 1 and Act d 8, occurred clearly less often. To assess possible explanations for this, the group of PR-10 sensitized subjects (*n* = 228) was split into two groups, based on this difference in the extent of PR-10 sensitization. Subjects that were sensitized to one or more ‘less common PR-10 proteins’ (*n* = 147; 64%) were compared with the ones only sensitized to one or more ‘common PR-10 proteins’ (*n* = 81; 36%). Several differences in sensitization patterns could be observed. In the group sensitized to the less common PR-10s, ISU values of all PR-10 proteins were significantly higher (all *p* < 0.0001; for example Bet v 1 median 28 [IQR 15–54] vs 8.0 [IQR 3.1–17.5]). Additionally, this group demonstrated more frequent sensitization to Phl p 6 (timothy grass; *p* < 0.0001) and Ole e 1 (olive pollen; *p* < 0.0001). Sensitization to the other components did not significantly differ in frequency or ISU levels but the total amount of (both food and inhalant) components recognized on the ISAC was significantly higher (median 22 vs 13 out of 112, *p* < 0.0001) in the group sensitized to uncommon PR-10s. Age and sex distribution was equal in the two groups.

### Co-sensitization within PR-10 and storage proteins

Thirty-nine percent of the food sensitized subjects (*n* = 265) was solely sensitized to food components of the PR-10 protein family and not to any of the other food protein families or components. Co-sensitization to multiple components of the PR-10 protein family was common (Fig. 3a). This co-sensitization was accompanied by a high correlation between the ISU values found for these components (Fig. 3b).

**Fig. 4:**
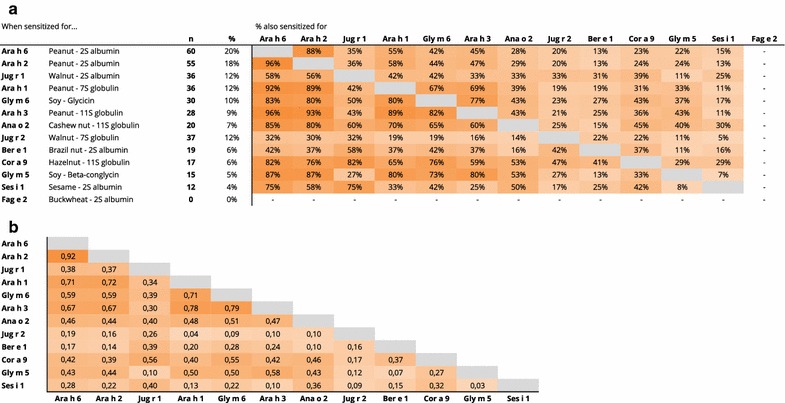
**a** Frequency of sensitization to storage proteins (left) and percentage of sensitized subjects with concomitant co-sensitization to other storage proteins (right). Darker shade of orange indicates higher frequency of co-sensitization. **b** Spearman correlation results of the ISU values found for the different storage proteins proteins

Within storage proteins, co-sensitization was limited (Fig. 4a). Co-sensitization was mostly observed between the different peanut storage proteins (Ara h 1, 2, 3, and 6) and, to a lesser extent, between Ara h 1 and 3 on the one hand and Gly m 6 (soy) and Ana o 2 (cashew) on the other hand. Of the four peanut storage proteins on the ISAC, subjects were on average sensitized to 3 out of 4 and the majority was sensitized to all four (100 out of 179 sensitized to peanut storage proteins). Subjects sensitized to Cor a 9, Gly m 5 and Ses i 1 were frequently co-sensitized to peanut, soy and/or walnut storage proteins. In our cohort, we observed mostly very low correlations in ISU values between the storage proteins (Fig. 4b). A clear exception was Ara h 2 and 6 (spearman’s rho 0.92; *p* < 0.001). Additionally, a strong correlation in ISU value was observed between Ara h 1 and 3, as well as Ara h 3 and Gly m 6.

## Discussion

In our study, sensitization to birch pollen related (PR-10) food allergens was very common, which is as expected in our birch-endemic area. Birch pollen’s Bet v 1 was overall the most recognized component with the highest median titer, supporting its assumed role as primary sensitizer [22]. Although co-sensitization to PR-10 proteins was very prevalent, sensitization frequency differed between food PR-10 proteins. When comparing the subjects sensitized to ‘uncommon PR-10 proteins’ from soy (Gly m 4), celery (Api g 1) and kiwi (Act d 8) with those sensitized to only the ‘common’ PR-10 proteins, we observed significantly more frequent sensitization to specific grass and olive tree pollen in the group sensitized to uncommon PR10 s as well as overall more extensive sensitization to components on ISAC. An interaction or causal relationship between these allergens is unlikely. These differences in the amount of PR-10 proteins recognized, could be due to variability in allergen exposure, structural homology or allergenic properties (e.g. lability) between Bet v 1 and other PR-10 proteins [23]. For example, Cor a 1.04 (hazelnut) and Mal d 1 (apple) both have 67% amino acid sequence identity with Bet v 1 [24, 25], while for Ara h 8 (peanut) and Api g 1 (celery) this is only 46 and 42% respectively [26]. Interestingly, for Act d 8 (kiwi) the sequence identity was 53%, which would rule out sequence identity as sole explanation [27]. Also, technical issues with (the components of) the ISAC platform could play a role in explaining the found differences in sensitization frequency. Sensitization to these ‘uncommon PR-10 proteins’ could be an indication of more extensive polysensitization in our Western European geographic area. Interestingly, a very similar ‘hierarchical order’ of sensitization frequency for PR-10 proteins (i.e. Bet v 1 > Cor a 1.04 > Mal d 1 etc.) was recently described in both Swedish children and Austrian adolescents, also characterized using multiplex specific IgE testing [28, 29]. While these are all birch endemic countries, overall sensitization patterns differed, especially in Austria where grass sensitization was more prevalent [29]. This could indicate that this hierarchical order of sensitization to PR-10 proteins is not influenced by co-sensitization to other types of allergens. Additionally, in the Swedish children, all part of the BAMSE birth cohort, this hierarchical order already revealed itself at the age of 4 years old and appeared to have stabilized around 16 years old [28].

We identified seven subjects sensitized to food PR-10 proteins, without concomitant sensitization to PR-10 proteins from pollen, present on the ISAC. Birch pollen’s Bet v 1 is considered the major PR-10 protein and primary sensitizer in birch pollen related food allergy [22]. Food PR-10 proteins are thought to be unable to induce primary sensitization due to their susceptibility to gastrointestinal digestion [30]. However, the co-sensitization to other peanut allergens in the Ara h 8 positive and pollen negative subjects could indicate primary sensitization to Ara h 8 via peanut ingestion or possibly skin contact [31]. Alternatively, technical aspects of the ISAC and its recombinant PR-10 components could have resulted in false positive or false negative results for food or pollen PR-10 proteins respectively. Other studies are needed to further investigate this. In literature, sensitization to PR-10s from hazelnut (Cor a 1) and peach (Pru p 1), without concomitant sensitization to Bet v 1, has been reported before [29, 32, 33]. Datema et al. [33] hypothesized that sensitization to Bet v 1-like pollen components from other *Fagales* species, such as hazel, oak, alder, or beech might be responsible for cross-reactive sensitization to Cor a 1 in their Bet v 1-negative patients. In our seven patients, alder’s Aln g 1 and hazel’s Cor a 1.01 were negative as well. Additionally, in our study, hazelnut Cor a 1.04 and apple Mal d 1 were recognized more frequently and with a higher median titer than alder’s Aln g 1 and hazel’s Cor a 1.01. Therefore, the sensitization potential of PR-10 proteins from pollen other than Bet v 1 appears limited. Still, Bet v 1-like pollen components from *Fagales* species have been identified, such as Que a 1 (*Quercus alba*; white oak), Cas s 1 (*Castanea sativa*; chestnut) and Fag s 1 (*Fagus sylvatica;* European beech), which could have played a role in these patients [34]. These components could not be tested in our population.

For storage proteins, cross-reactivity between proteins from different foods is uncommon [35]. However, we did observe some striking combinations in co-sensitization in our population. For peanut allergens, co-sensitization to multiple peanut proteins was common. While Ara h 2 and 6 are both 2S albumins, Ara h 1 and 3 belong to different subtypes of peanut storage proteins. Cross-reactivity between Ara h 2 and 6 has been demonstrated in several experiments [36–38], but there is limited data suggesting cross-reactivity between Ara h 2 on the one hand and Ara h 1 and 3 on the other hand [36]. In our data, we observed a moderately strong correlation in ISU levels between Ara h 1 and 3 and between those two and Ara h 2 and 6, which could be the result of common co-sensitization as well as cross-reactivity. Another common co-sensitization was between peanut and soy components. This was also observed before in a population of soy allergic patients from our clinic [39]. While the moderately strong correlations suggest potential cross-reactivity, inhibition experiments are needed to confirm this finding. Interestingly, sensitization to less frequently recognized allergens cashew Ana o 2, hazelnut Cor a 9, soy Gly m 5 and sesame Ses i 1 was often accompanied with co-sensitization to peanut. In the case of Cor a 9 and Ses i 1, co-sensitization to walnut Jug r 1 was common as well. While these observations could indicate distinct patterns in sensitization, they might be (partly) influenced by patient selection for ISAC testing. Different studies are needed to properly assess the clinical relevance of these co-sensitizations [18].

The ISACs in this population were ordered during routine care in outpatients with a suspected food allergy. While it is therefore an unselected outpatient group, there is a certain selection bias due to the nature of the test. The ISAC consists of a selection of food components, mostly of plant food origin. However, certain components that have been established to be clinically relevant, such as hazelnut Cor a 14 [10], are not present on the ISAC. Additionally, most components are also available separately on the ImmunoCAP platform, making it financially unfeasible to perform an ISAC in case of few suspected allergies, e.g. only peanut, a single tree nut or isolated fish or shellfish allergy. Due to the selection of components on the ISAC, together with the financial costs of the test, in our tertiary food allergy clinic, the ISAC is generally ordered for suspected multiple plant food allergies. Additionally, sensitization to nsLTPs and profilins is infrequent in our population and its role in clinical allergies in our population appears limited [10, 40, 41]. Therefore, we chose to focus this study on PR-10 and storage proteins. Another important limitation of this study is the use of sensitization data, without data on food allergy diagnosis, clinical symptoms or atopic co-morbidity. Clinically irrelevant sensitization is a known limitation of sensitization tests in (food) allergy diagnostics, especially with cross-reactive allergens such as PR-10 proteins [42, 43]. Furthermore, we did not take potential seasonal variation in (specific) IgE levels into account [44, 45], although this effect has not been studied with ISAC yet. Strength of this study is the analyses of a large adult population using a current multiplex specific IgE test with 112 components. Similar studies were mainly focused on children or adolescents [29] or performed using older test platforms with less components [46].

## Conclusions

In our population of adults with a suspected food allergy, sensitization to PR-10 proteins was most frequent, often with co-sensitization to food allergens from other protein families. Within the storage proteins, sensitization to multiple peanut allergens was most common (on average 3 out of 4). Sensitization to PR-10 proteins from food, without concomitant sensitization to PR-10 proteins from pollen, occurred in a subset of subjects. Less commonly recognized PR-10 proteins appeared indicative of polysensitization to both food and inhalant allergens.

## Additional file

**Additional file 1: Table** **1.** All components present on the ImmunoCAP ISAC 112, with their sensitization frequency and median ISU value in the total population (*n* = 305).

## Abbreviations

CRD: component-resolved diagnostics
ISAC: ImmunoCAP ISAC
ISU: ISAC Standardized Units
PR-10: pathogenesis related protein family 10
nsLTP: non-specific lipid transfer protein
sIgE: specific IgE
