# Supplementary material for: Sensitization to PR-10 proteins is indicative of distinctive sensitization patterns in adults with a suspected food allergy
Source: Clin Transl Allergy. 2017 Nov 23;7:42. doi: 10.1186/s13601-017-0177-4 (PMC5700688; doi:10.1186/s13601-017-0177-4)
Supplement: Supplementary file 1 — Additional file 1: Table 1. All components present on the ImmunoCAP ISAC 112, with their sensitization frequency and median ISU value in the total population (n = 305). [file 13601_2017_177_MOESM1_ESM.pdf]

Additional file 1: all components present on the ImmunoCAP ISAC 112, with their sensitization frequency and median ISU value in the total population (n=305)

Component	Source	Allergen type	Sensitization frequency (%)	Median ISU value
Bet v 1	Birch pollen	Inhalant	72,5	19,4
Cor a 1.04	Hazelnut	Food	72,1	6,7
Mal d 1	Apple	Food	67,9	7,5
Aln g 1	Alder pollen	Inhalant	67,9	6,2
Cor a 1.01	Hazel pollen	Inhalant	65,6	4,3
Phl p 1	Timothy pollen	Inhalant	63,0	12,5
Pru p 1	Peach	Food	62,3	4,0
Cyn d 1	Bermuda pollen	Inhalant	57,4	5,1
Ara h 8	Peanut	Food	53,8	1,9
Fel d 1	Cat	Inhalant	46,9	6,1
Phl p 5	Timothy pollen	Inhalant	45,2	9,6
Der f 2	Dermatophagoides mite	Inhalant	44,6	14,7
Der p 2	Dermatophagoides mite	Inhalant	43,3	9,7
Phl p 4	Timothy pollen	Inhalant	42,0	2,7
Der f 1	Dermatophagoides mite	Inhalant	39,3	7,4
Der p 1	Dermatophagoides mite	Inhalant	38,4	7,1
Gly m 4	Soy	Food	37,7	1,2
Phl p 2	Timothy pollen	Inhalant	33,1	5,0
Phl p 6	Timothy pollen	Inhalant	32,5	1,8
Ole e 1	Olive pollen	Inhalant	29,8	5,2
Api g 1	Celery	Food	26,9	1,7
Act d 8	Kiwi	Food	21,3	0,7
Can f 5	Dog	Inhalant	20,7	2,0
Can f 1	Dog	Inhalant	20,3	5,6
Ara h 6	Peanut	Food	19,7	6,5
Ara h 2	Peanut	Food	18,0	4,9
Equ c 1	Horse	Inhalant	17,4	2,6
Mer a 1	Mercury pollen	Inhalant	16,7	2,6
Hev b 8	Latex	Other	16,1	2,8
Cup a 1	Cypress pollen	Inhalant	15,4	1,0
Bet v 2	Birch pollen	Inhalant	14,8	2,3
Phl p 11	Timothy pollen	Inhalant	14,4	6,5
Fel d 4	Cat	Inhalant	13,8	2,9
Phl p 12	Timothy pollen	Inhalant	13,1	1,1
Mus m 1	Mouse	Inhalant	12,1	3,8
Ara h 1	Peanut	Food	11,8	10,6
Jug r 1	Walnut	Food	11,8	3,8
Lep d 2	Lepidoglyphus mite	Inhalant	11,5	1,5
Pla a 2	Plane pollen	Inhalant	10,8	0,7
Jug r 3	Walnut	Food	10,5	1,1
MUXF3	Bromelin	Other	10,5	0,6
Alt a 1	Alternaria	Inhalant	9,8	3,0
Gly m 6	Soy	Food	9,8	3,4

Component	Source	Allergen type	Sensitization frequency (%)	Median ISU value
Ara h 3	Peanut	Food	9,2	6,9
Cry j 1	Japanese cedar pollen	Inhalant	9,2	0,8
Can f 2	Dog	Inhalant	8,2	4,3
Act d 2	Kiwi	Food	7,9	0,8
Asp f 6	Aspergillus	Inhalant	7,5	3,0
Art v 1	Mugwort pollen	Inhalant	7,5	2,5
Act d 1	Kiwi	Food	7,2	2,6
Der p 10	Dermatophagoides mite	Inhalant	7,2	0,8
Blo t 5	Blomia mite	Inhalant	6,9	2,8
Ana o 2	Cashew nut	Food	6,6	1,4
Ber e 1	Brazil nut	Food	6,2	0,7
Fel d 2	Cat	Inhalant	6,2	2,0
Jug r 2	Walnut	Food	6,2	0,5
Can f 3	Dog	Inhalant	5,9	2,8
Gal d 3	Egg white	Food	5,9	1,2
Pru p 3	Peach	Food	5,9	1,3
Cor a 9	Hazelnut	Food	5,6	0,9
Pen m 2	Shrimp	Food	5,6	3,0
Bos d 8	Cow's milk	Food	5,2	6,6
Ole e 9	Olive pollen	Inhalant	5,2	0,7
Gal d 1	Egg white	Food	5,2	3,6
Hev b 6	Latex	Other	5,2	6,9
Che a 1	Pigweed pollen	Inhalant	4,9	0,5
Asp f 3	Aspergillus	Inhalant	4,9	1,4
Gly m 5	Soy	Food	4,9	1,3
Pla a 3	Plane pollen	Inhalant	4,9	0,8
Ves v 5	Common wasp	Other	4,9	0,6
Bos d 4	Cow's milk	Food	4,6	1,0
Cor a 8	Hazelnut	Food	4,6	0,8
Bos d 5	Cow's milk	Food	4,3	4,2
Gal d 2	Egg white	Food	4,3	4,1
Bla g 7	Cockroach	Inhalant	3,9	13,3
Ses i 1	Sesame	Food	3,9	2,7
Ara h 9	Peanut	Food	3,6	1,4
Art v 3	Mugwort pollen	Inhalant	3,6	1,5
Equ c 3	Horse	Inhalant	3,6	1,7
Pen m 1	Shrimp	Food	3,6	10,4
Phl p 7	Timothy pollen	Inhalant	3,6	9,7
Ani s 3	Anisakis	Other	3,3	17,6
Bet v 4	Birch pollen	Inhalant	3,3	6,6
Alt a 6	Alternaria	Inhalant	3,0	1,1
Bos d 6	Cow's milk & beef	Food	3,0	1,2
Gad c 1	Cod	Food	3,0	19,5
Pen m 4	Shrimp	Food	2,6	1,1
Asp f 1	Aspergillus	Inhalant	2,3	1,0

Component	Source	Allergen type	Sensitization frequency (%)	Median ISU value
Par j 2	Wall pellitory pollen	Inhalant	2,3	0,8
Pol d 5	Paper wasp	Other	2,3	1,2
Tri a 14	Wheat	Food	2,3	1,6
Bla g 2	Cockroach	Inhalant	2,0	0,4
Gal d 5	Egg yolk/chicken	Food	2,0	8,7
Api m 4	Honey bee	Other	1,6	0,6
Cla h 8	Cladosporium	Inhalant	1,6	0,8
Ole e 7	Olive pollen	Inhalant	1,6	0,8
Bos d lactoferrin	Cow's milk	Food	1,3	0,6
Hev b 5	Latex	Other	1,3	3,9
Pla a 1	Plane pollen	Inhalant	1,3	0,7
Tri a 19.0101	Wheat	Food	1,3	2,3
Tri a aA TI	Wheat	Food	1,3	0,7
Hev b 1	Latex	Other	1,0	2,6
Hev b 3	Latex	Other	1,0	3,1
Pla l 1	Plantain	Inhalant	1,0	6,0
Sal k 1	Saltwort	Inhalant	1,0	1,5
Api m 1	Honey bee	Other	0,7	0,5
Amb a 1	Ragweed	Inhalant	0,3	1,0
Ani s 1	Anisakis	Other	0,3	0,3
Bla g 1	Cockroach	Inhalant	0,3	0,3
Act d 5	Kiwi	Food	0,0	0,0
Bla g 5	Cockroach	Inhalant	0,0	0,0
Fag e 2	Buckwheat	Food	0,0	0,0
